# Anticipatory Regimes in Pregnancy: Cross-Fertilising Reproduction and Parenting Culture Studies

**DOI:** 10.1177/00380385221107492

**Published:** 2022-08-07

**Authors:** Edmée Ballif

**Affiliations:** University of Cambridge, UK; University of Kent, UK

**Keywords:** anticipation, foetus, parenting, pregnancy, reproduction, risk, Switzerland, temporality, time

## Abstract

Despite attempts at highlighting continuities across the reproductive process from conception to childcare, reproduction and parenting still tend to be studied as a collection of separate objects. This article contributes to the cross-fertilisation of reproductive and parenting culture studies by first introducing anticipation as a transversal analytical lens. A conceptual framework for the analysis of anticipatory regimes in reproduction is introduced with a focus on subjectification effects and future images. Second, the importance of pregnancy as a connector between reproduction and parenting is highlighted. These propositions are fleshed out with reference to an ethnography of pregnancy care in Switzerland. The results demonstrate that pregnant women are expected to act as anticipating agents and that foetuses are treated as future children. Future images reveal how prenatal care reproduces gender norms. Analysing anticipatory regimes contributes to discussions of power relations in prenatal care, the stratification of reproduction and challenges to reproductive justice.

## Introduction

As Lupton (1999: 59–60) summarised, ‘at the end of the twentieth century the pregnant woman is surrounded by a complex network of discourses and practices directed at the surveillance and regulation of her body’ – an observation that remains true today. Medical advice and popular discourses urge pregnant women to autoregulate their bodies carefully to protect their foetuses. This trend has not escaped the attention of feminist academics, who have critically analysed the responsibilisation of pregnant women and its consequences for reproductive autonomy (see, for example, Armstrong and Abel, 2000; Bell et al., 2009; Fixmer-Oraiz, 2019; Kukla, 2010; Lowe and Lee, 2010; Ruhl, 1999; Weir, 1996). Parenting culture scholars have interpreted this trend as ‘extending parenting culture backwards’ (Lee et al., 2010: 297) to prospective parents, whereby the increasingly intensive expectations upon parents colonise the period before birth (Lee et al., 2014a). Pregnancy appears to have become part of parenting, blurring the line between born and unborn children, between antenatal and postnatal subjects (Weir, 2006). The regulation of pregnancy also echoes the social control of reproductive bodies more generally (Briggs, 2017; Duden, 1993; Martin, 1992). With the emergence of pre-pregnancy care in the USA, women are now urged to adopt risk-reducing behaviours even before getting pregnant, which positions some bodies as always potentially pregnant (Waggoner, 2017). In spite of this, reproductive stages before and after pregnancy still tend to be treated as separate fields of research.

Faircloth and Gürtin (2018) called for a more comparative approach across reproductive events and identified overarching themes at the intersection of reproduction and parenting (reflexivity, gender, expertise and stratification). Building on this transversal perspective, I make two further contributions to the dialogue between reproductive and parenting culture studies in this article. First, I introduce anticipation as another overarching theme across the reproductive process. Anticipation underlies a vast array of practices and observed trends in reproduction and parenting in Europe and in the USA,^1^ despite rarely been explicitly analysed as such. I introduce a conceptual framework that focuses the analysis of anticipatory regimes in two dimensions: subjectification effects (the constitution and positioning of subjects in discourse) and future images (representations of a good or bad future).

Second, I argue that pregnancy is a particularly fertile ground on which to highlight continuities across the reproductive process. Some scholars have demonstrated the relevance to pregnancy of concepts often discussed in reproductive or parenting culture studies such as risk, medicalisation and surveillance (Barker, 1998; Lee et al., 2014a; Lupton, 1999; Ruhl, 1999; Weir, 2006). Yet pregnancy, despite being a fairly common experience, has not been studied as much as other events in the reproduction–parenting spectrum such as fertility, abortion, birth or childcare (Ivry, 2015; Teman and Ivry, 2021), with a few notable exceptions (Han, 2013; Ivry, 2010; Kukla, 2005). By asking, ‘What are the effects of anticipation in the government of pregnancy?’, this article illustrates the potential of anticipation as an analytical concept and of pregnancy as a site to think across reproduction and parenting.

I flesh out these arguments using an ethnography of pregnancy care in Switzerland, from which the outlined arguments originate. I conducted fieldwork in a state-funded unit that provides information and support to pregnant women^2^ on the ‘psychosocial’ level, in the form of free appointments to discuss health-related, emotional or social aspects of pregnancy (Ballif, 2017). The unit is part of a larger public health programme aimed at promoting health among children under school age. Unlike most types of family surveillance (Donzelot, 1977; Hardyment, 2007; Ladd-Taylor and Umansky, 1998), the Perinatal Unit (PU)^3^ constitutes a rare but illustrative form of social surveillance and guidance of families *before* birth. The PU thus represents an ideal site to observe overarching norms and representations in both reproduction and parenting. Analysing the effects of anticipation in this context resulted in the identification of the ‘anticipating pregnant woman’ and the ‘anticipated foetal subject’ as the main subjects in pregnancy counselling.

## Anticipation in Reproduction and Parenting

Anticipation can be defined as a regime of temporality where the present is always thought of and acted upon in reference to possible futures (Adams et al., 2009; Anderson, 2010; Tavory and Eliasoph, 2013). It is a component of contemporary subjectivities (a way of thinking, feeling and acting) as well as a dimension of governmentality and political practice (Adams et al., 2009; Anderson, 2010). Policies and political programmes in education or the environment for example have been analysed as anticipatory regimes since they set out a plan for action in the present to enable certain futures (Amsler and Facer, 2017; Groves, 2017). As such, anticipation is a key component of Giddens’ (1991) and Beck’s (1992) theories of modernity that describe individuals as dealing with more and more uncertainty in a ‘risk society’. Risk has indeed been described as a tool to ‘discipline’ (Ewald, 1991: 207), ‘colonise’ (Giddens, 1991: 129) or ‘control’ (Rose, 2007: 70) the future in the present.

Risk is a central theoretical component of parenting culture studies, and scholars have argued that, in recent decades, child-rearing has been transformed in a way that centres risk and uncertainty (Hays, 1996; Lee et al., 2014a). Indeed, one of the main characteristics of contemporary reproduction and parenting regimes is the assumption that ‘the unborn’ (i.e. embryos and foetuses), infants and children are vulnerable entities who must be protected in order for them to develop safely (Armstrong, 2003; Lupton, 2013; MacKendrick and Cairns, 2019; Oaks, 2001). Parents bear the responsibility of monitoring and enabling the best possible outcomes for their children, resulting in the contemporary ‘labor-intensive’ parenting culture (Hays, 1996: 8). In other words, a specific anticipatory ethos lies at the heart of contemporary parenting culture, relying on risk-consciousness and awareness that something may always go wrong.

Temporalities, including anticipatory logics, are an emerging theme in reproductive studies (Beynon-Jones, 2017; Dow, 2016; Franklin, 2014; Sänger, 2015). At the core of reproduction lies a relationship with the future, over which people attempt to gain control. According to recent studies of assisted reproduction for example, prospective beneficiaries of reproductive technologies usually face a selection or screening process whereby their right to access the desired reproductive process is contingent upon an evaluation of the welfare of the future child (Bühler, 2021; De Jong, 2015; Lee et al., 2014b).

The ‘government of pregnancy’ (Ballif, 2020; Weir, 1996) illustrates the dominance of anticipatory regimes in the reproductive process. Borrowing from Foucault’s (2007) theory of a ‘government of life’, with the ‘government’ of pregnancy, I invoke the networks of power relations and knowledge aimed at regulating pregnancy. Prenatal care, midwifery or public health, for example, operate both at the individual level (as ‘disciplines’) and at the population level (as ‘governmentalities’ or ‘biopolitics’) to ensure the life and health of society members (Foucault, 1990). Sociological and anthropological studies of pregnancy in recent decades have illustrated – but rarely explicitly acknowledged – that anticipation is a central component of the way pregnancies are ‘governed’. Historically, antenatal care was developed with a preventative goal: to detect and treat diseases or complications like syphilis or preeclampsia in order to avoid bad outcomes for the foetus or the mother (Hanson, 2004; Oakley, 1984; Wertz and Wertz, 1977). Contemporary pregnancy experiences have been analysed in terms of medicalisation and risk management – both trends being premised upon the goal of acting in the present to avoid future complications (Barker, 1998; Lyerly et al., 2009; Ruhl, 1999; Weir, 2006). Foetuses have emerged as targets of political, medical and social interventions in the interest of their future existence as individuals (Casper, 1998; Franklin and Ginsburg, 2019; Hartouni, 1992; Petchesky, 1987). The government of pregnancy thus provides an excellent site to observe regimes of anticipation in reproduction and parenting, as a reproductive event spanning across the traditional limits of the two academic fields.

I suggest that at least two dimensions of anticipation are particularly relevant for this field. First, *subjectification effects* of anticipatory regimes deserve attention; that is, how subjects are constituted and positioned in discourses (Bacchi, 2009; Foucault, 1982). What consequences do anticipatory regimes have on the status, rights and responsibilities of subjects? How are individuals expected to act as anticipating agents? Examples of such subjects include the ‘pregnant addict’, ‘drinker’ or ‘smoker’, who are all figures of bad mothering because of the long-lasting harm they are considered to cause to their offspring (Ballif, 2019; Humphries et al., 1992; Lee, 2014; Oaks, 2001). How pregnant subjects are positioned, how their autonomy is framed as entire or limited in consideration to the foetus, how foetuses are positioned as political subjects in their own rights or not – all of these discursive elements have concrete (and sometimes life-threatening) consequences on access to health care and on reproductive justice (Luna, 2020; Ross and Solinger, 2017).

Second, *future images* involved in anticipatory regimes should be analysed. By ‘future images’, I mean the representations of ‘future horizons’ (Groves, 2017: 31), either desired or feared, that anticipation conjures. For example, ‘ordinary’ experiences of pregnancy (Han, 2013) such as taking ultrasounds, purchasing advice literature or choosing childcare can be driven both by hopes of a good future (such as, raising a healthy child) and anxiety over undesired outcomes. Future images are important because they reveal social norms and hierarchies. Population control and eugenics, for instance, aim to bring about collective futures that are considered more desirable, either by eliminating categories considered unfit or by encouraging the reproduction of other groups deemed worthy along race and class lines (Gerodetti, 2006; Mottier, 2008). Analyses of contemporary reproductive politics illustrate how the control of reproductive lives relies on hopes and fears about the future that are racialised, gendered and sexualised (Briggs, 2017; Franklin and Ginsburg, 2019).

The analysis of subjectification effects and future images could form the basis for comparisons across reproductive stages and across time and space. The rest of this article illustrates the potential and relevance of this approach by drawing upon an ethnographic case study of a pregnancy care unit in Switzerland.

## Governing Pregnancy at the Perinatal Unit

The Swiss context is characterised by a long history of state interventions into reproductive matters. Switzerland was at the forefront of eugenic policies in the 19th and 20th centuries (Jeanmonod and Heller, 2001; Mottier, 2005; Wecker, 1998). Resistance to reproductive rights is still strong and reveals the persistence of conservative family ethics. Abortion has only been legal at the federal level since 2002. Access to assisted reproductive technology (ART) is restricted, the process is expensive and egg donation and surrogacy are prohibited.

Against this backdrop, I chose the case of the PU in French-speaking Switzerland because of its unusual ‘psychosocial’ orientation to pregnancy care. In a context where pregnancy care and birth are highly medicalised,^4^ the PU’s focus on the psychological, financial and relational aspects of pregnancy signals a shift beyond the ‘medicalisation’ of pregnancy towards a more global surveillance of pregnant women and parents (Ballif, 2017, 2020). Created in 1986, its services are aimed at the general population, and not specifically at poorer or racialised families, illustrating a growing tendency to problematise all forms of parenting, including in privileged middle-class families (Lee and Macvarish, 2020). At the time of the study, the PU’s midwives and social workers met 2400 future parents per year, a number that corresponded to a quarter of all pregnancies in the region. The PU was visited almost exclusively by heterosexual, cisgender women and, in one in four consultations, their male partners – despite a declared LGBTQ+- and men-friendly policy.

I conducted ethnographic fieldwork in the PU over 22 months between 2011 and 2013. I followed an inductive research design to allow for the emergence of new, in-context insights into this under-researched field (Glaser and Strauss, 1967). I sought to understand the PU as a site where professionals transmit ‘a specific *technic* of pregnancy’ (Kukla, 2005: 128, emphasis in original) to pregnant women. This implied a focus on professionals’ perspectives rather than patients’, in contrast with earlier studies on women’s experiences of prenatal care (Bessett, 2010; Browner and Press, 1996; Hammer and Burton-Jeangros, 2013). Having worked as a part-time administrative assistant there a few years earlier helped me to gain access to the setting, as did the fact that I was, like all staff members, a white, cisgender, heterosexual woman. I observed and took notes during appointments with pregnant women (n = 29) or couples (n = 2) and during 60 staff meetings (continuing education, administrative organisation, team building or case discussions). Staff members and clients gave their informed consent before the start of the observed sessions and were guaranteed confidentiality.^5^ I conducted semi-structured interviews with each of the 18 staff members (55 to 140 minutes) to explore the various meanings attached to pregnancy counselling. Questions covered participants’ career paths (education, motivations and reasons for choosing to work in the PU) and conceptualisations of their roles as midwives or social workers (meanings attached to their jobs, values, etc.). Interviews were audio-recorded and transcribed; handwritten notes were taken where recording was refused. I also collected PU policy documents and internal reports.

All data were coded using a spreadsheet to facilitate the identification of recurring themes. Drawing from poststructuralist discourse and policy analysis methods (Arribas-Ayllon and Walkerdine, 2008; Bacchi, 2012; Hall, 2001; Larsson, 2018), data analysis aimed at identifying ‘problematisations’ as well as ‘subjectification effects’ in PU staff’s discourses (Foucault, 1982, 1988).

It is in the course of this analysis that anticipation emerged as a topic underlying pregnancy counselling. Overall, the results suggested that pregnancy government in the PU relied on the problematisation of pregnancy as a period of vulnerability that requires active self-management and expert guidance. In what follows, I focus on data that relate to anticipation with regards to the two main subjects discussed during PU appointments: the pregnant woman and the foetal subject. Other figures such as fathers, partners, siblings or grandparents were less often discussed, which is typical of the dominant forms of pregnancy care as focused on the unborn–maternal assemblages (Daniels, 1999; Lupton, 2013).

## The Anticipating Pregnant Subject

The anticipatory dimension of pregnancy counselling first became apparent as I was observing consultations at the PU. Midwives and social assistants structured their discussions with pregnant women according to a patient interview guide. Along with the typical prenatal care questions (due date, maternal health, medical history), the interview guide included obstetrical history (abortions or miscarriages), alcohol and tobacco consumption, domestic violence history and relationship status (with the future father and/or the woman’s partner). A risk logic underlies these questions (Ballif, 2014): depending on the pregnant women’s responses to these questions, midwives and social workers gave advice to adopt particular risk-reducing behaviours (e.g. by reducing their alcohol intake) or suggested a follow-up appointment. A social worker explained to me that abortions, past depressive episodes or relationship problems were also considered risk factors in the PU, since these women were considered more at risk of a postpartum psychological ‘breakdown’. Deploying this interview guide thus formed part of a disciplinary regime wherein staff anticipated the possible futures of each patient, weighed them against their (normative) definition of desirable outcomes and suggested actions.

As a subjectification effect of this anticipatory regime, PU appointments positioned pregnant women themselves as self-regulated, anticipating subjects: they were expected to think and act in anticipation to expected future events. Some interview questions explicitly assessed pregnant women’s future: birth choices (choice of birth centre, birth plan), organisation of maternal leave and return to work, choice of paediatrician and childcare arrangements. During interviews with me, staff explained that these questions were intended to evaluate future parents’ ‘preparedness’. Anticipation was thus a moral imperative for pregnant women, one that goes along with already identified imperatives of responsibility, self-discipline and self-sacrifice of the pregnant subject (Lupton, 1999; Marshall and Woollett, 2000; Ruhl, 1999).

Pregnant women’s degree of preparedness was evaluated according to specific future images. For example, Catherine, a social worker at the PU, told me about a woman she thought was not anticipating her future in a ‘realistic’ way:

The other day, I received a 26-year-old woman who wants to start studying midwifery in September although she will give birth in December, and on top of that she will take care of her partner’s two children. She is in denial! (Fieldnotes, June 2013)^6^

Catherine’s qualification of this woman’s plans as ‘denial’ implies that anticipation must be indexed on a normative ‘reality’: a less ambitious conciliation of career and parenting, if not a complete relinquishment of professional plans in favour of mothering. Here, ‘failed’ anticipation reveals the normalising and moralising definition of the ‘proper’ anticipating pregnant subject. Although implicitly, midwives and social workers’ discourses on anticipation and preparedness revealed deeply rooted representations of ‘proper’ life with a baby as the bourgeois, middle-class, white and heteronormative model of the family. These assumptions are at odds with the self-presentation of the PU staff as progressive, leftist, feminist and open to all family models.

Another example of failed anticipation happened during the consultation between Agnes, a social worker, and Mrs Fischer, a 34-year-old married woman who was due to give birth to her first child two weeks later. During their first appointment, Mrs Fisher asked if her husband could take a paternal leave and stay with the baby because she wanted to go back to work immediately at the end of her 14-week maternity leave (the legal minimum in Switzerland). Visibly surprised at this plan, Agnes did not answer Mrs Fischer’s precise question but laughed and continued the conversation as such:

Agnes: Have you already taken care of other babies?

Mrs Fischer: A little. Not really. I don’t have a lot of experience, but I know a lot of things.

Agnes: Yes but when I hear you, I think ‘She is not in Baby’s^7^ reality!’ (laughs)

Mrs Fischer: Oh, yes?

Agnes: Because you are already thinking about going back to work, I thought, ‘Oh gosh, when Baby’s here, I would be curious to see what you think!’

Mrs Fischer: No, I know it will be difficult; I hear mothers talk-

Agnes: It’s hard to leave them to go to work! Because sometimes you have plans and when the baby is here, you think ‘Can I really do this, what are my priorities . . .?’ It changes. This is why I think it’s good to know your options but leave decisions for after the birth.

Mrs Fischer: I think so too. Maybe I will change my mind. (Fieldnotes, June 2013)

Agnes hardly hid her disapproval of Mrs Fischer’s plans. Again, the future anticipated by the pregnant woman was considered not to be in line with the ‘reality’ of having a baby. Here, this ‘reality’ entails, in Agnes’ view, a shifting of priorities from work to mothering. Since this shift appears not to have happened in Mrs Fischer’s case, Agnes explains in a rather patronising tone that she should wait until it does before making any decisions. In her electronic file (inaccessible to Mrs Fischer), Agnes wrote: ‘Mrs Fischer does not seem aware of the baby’s arrival.’ The vocabulary of ‘reality’ and ‘awareness’ bluntly revels the uncritical normative representation of a pregnant woman’s future as prioritising maternity over career. This happens in the Swiss context where women assume the vast majority of domestic labour and childcare duties, which translates into one of the highest rates of part-time employment among women in European comparison (Swiss Federal Statistical Office, 2020).

Consultations at the PU thus contribute to the formation of anticipating pregnant subjects that should adopt ‘proper’ maternal behaviour in advance – a perfect illustration of the extension of parenting culture backwards (Lee et al., 2010). The emergence of such an anticipating regime in prenatal care offers insights into the way power relations operate in this field. It has often been noted how in governmentality, power operates ‘at a distance’, with individuals becoming involved in self-surveillance and self-regulation (Lupton, 1995; Murphy, 2003; Weir, 1996). Catherine’s and Agnes’ interventions illustrate a more direct disciplinary power, with individual pregnant subjects placed under scrutiny and compelled to change their behaviour. Pregnant subjects are not passive recipients of such power relations, and their strategies of resistance have been described elsewhere (Hammer and Burton-Jeangros, 2013; Hammer and Inglin, 2014; Root and Browner, 2001).

## The Anticipated Foetal Subject

What effect does anticipation have on representations of foetuses? Feminist analyses have often focused on pro-life discourses and highlighted how foetuses are positioned as political subjects deserving protection (Franklin, 1991; Hartouni, 1992; Morgan and Michaels, 1999; Petchesky, 1987). Anticipation clearly underlies such discourses that project foetuses as potential members of society. The social life of foetuses extends beyond abortion politics, however, as the treatment of unborn entities is politically and legally regulated in other, less-studied ways (Lupton, 2013).

The way foetuses are positioned in Swiss laws is a good illustration of the various ways foetuses can be represented: as ‘unborn’, as ‘persons’ or as ‘future children’. Even though foetal rights are not as intensely debated as in other national contexts such as the USA or Ireland, discussions surrounding the status of the unborn regularly come up in the context of direct democracy, like when laws on assisted reproduction, research, abortion or embryo genetic testing were submitted to popular vote over the past two decades.

First, the life of foetuses ‘as unborn’ entities is protected through the regulation of abortion, which is legal in Switzerland until 12 weeks of amenorrhea. Abortion based on medical indications (relative to physical or psychological distress) is possible after that time. In other words, after 12 weeks, foetuses ‘as unborn’ are protected by the state, which delegates to medical experts the right to decide to terminate a pregnancy. Second, foetuses are granted a person-like status in some domains (foetus as ‘person’). For example, the 2014 Human Research Act grants unborn entities the same legal protection as persons when it comes to scientific research: the law aims to safeguard the ‘dignity, privacy and health of people involved in research’ by regulating research on ‘persons, deceased persons, embryos and foetuses, biological material and health data’. In addition, since the 19th century, stillborn children (born after 22 weeks of pregnancy or which weigh more than 500 grams) must be registered with the civil registry. This means that, demographically, five-month-old foetuses are considered part of the population. Since 2019, a new legal provision allows parents to register ‘miscarried children’ (*Fehlgeburt*) regardless of their age or weight. Switzerland is not an exception in this regard (Austin and McGuinness, 2019; Middlemiss, 2021; Sénat français, 2008). In these two instances, foetuses are explicitly positioned on the same level and granted the same rights as living persons, in contrast to abortion laws, which do not make this explicit parallel.

Third, foetuses can be protected in the name of their potentiality: as ‘future children’ (Ballif, 2020). This is illustrated by the 1998 Reproductive Medicine Act, which sets the ‘well-being of the child’ as the first requirement for the use of ART. ART is thus reserved for ‘couples who, on the basis of their age and personal circumstances, are likely to be able to care for and bring up the child until it reaches the age of majority’, which in practice excludes single mothers and older prospective parents (Bühler, 2021; De Jong, 2015). The prohibition of ovum and embryo donation and surrogacy, as well as the limiting of access to sperm donation to ‘married’ couples further excludes single persons and same-sex couples (before 2022 at least, when same-sex marriage was legalised and allowed access to ARTs to married same-sex couples). Thus, this law projects even un-conceived entities into the future and seeks to ensure a ‘good’ future for them along sexual, gendered, class and moral norms. It is in this third subject position that an anticipatory logic is most obvious since ideas about the future life of foetuses are integrated into decision making in the present.

In the PU, foetuses were very rarely treated as ‘unborn’ entities in discussions: the words ‘embryo’ or ‘foetus’ were hardly used; I never saw an ultrasound image circulating, nor heard a professional enquire about foetal development, and neither did I ever hear a pregnant woman describe the feelings of foetal movements. This was probably due to the absence of physical examination or discussions about abortion in the PU. Moreover, foetuses were never discussed as full ‘persons’ or members of the family. Instead, foetuses were treated as ‘future children’, illustrating the importance of the anticipatory framework. The effect of anticipation is to extend children’s rights and status to foetuses, not because foetuses are regarded as children in their own right, but because eventually they *will be* children, or so it is conceived.

As part of a larger public health programme, the PU is dedicated to the promotion of *children*’s health and well-being through early intervention in pregnancy. This extension of children’s protection backwards into pregnancy is at odds with Switzerland’s legal framework since, technically, all children’s rights begin at birth. This tension is well present in the case of child abuse. In the canton where the PU operates, every professional working with minors has a legal obligation to report to child protection services any child ‘who appears to need help’. During a staff meeting, a social worker asked a lawyer whether such child welfare laws applied where the child was not yet born. The lawyer answered that, technically, child welfare laws only apply after birth since unborn children do not possess a legal personality: ‘If you report a child who seems to be in need of protection before birth, there is no legal basis for that’ (Fieldnotes, November 2012). Despite this, PU staff explained that, by virtue of a tacit agreement between institutions, they regularly reported unborn children to child protection services in their region. Both PU and child protection services considered that this ensured a speedy processing of cases. Occasionally, child protection services even met future parents before birth. Thus, in practice, unborn children *are* objects of protective measures by professionals and the state, just as future parents are objects of surveillance: the corollary to ‘parenting before children’ is ‘protecting children before birth’.

Turning to images of foetuses’ futures, the reproduction of social values becomes apparent. Just as pregnant subjects are expected to prepare for a future that conforms to dominant gender roles, midwives and social workers anticipate the future life of foetal subjects within a traditional family model. This is especially salient in their anticipatory treatment of child recognition. Until 2014, a child born in Switzerland to an unwed mother, and who was not officially recognised by a man was automatically placed under the state’s protection through a so-called ‘paternal guardianship’.^8^ An enquiry was then opened to identify the father. A variety of reasons can lead to the absence of paternal recognition: a mother could be unable or unwilling to contact the father (in the case of conflict, violence, sexual assault or in cases where there are multiple or unidentified sexual partners, for example), or a presumed father could be reluctant to proceed with official recognition (in order to avoid paying child support or in the case of doubts over paternity, for example). In practice, social services remained involved with unwed mothers for an average of three to four years, usually well beyond the time needed to establish paternity. Since 2014, paternal guardianship is no longer automatic but can still be implemented by child protection authorities (article 308 of the Swiss Civil Code). Single mother families are framed as a deficient family form, which needs to be managed by state authorities until a father can take over his role. In the PU, I heard social workers warning unwed mothers that paternal recognition was ‘obligatory’ (it is not) and that they should press the (presumed) father to step forward. During their discussions with me, social workers framed this as a children’s rights issue: ‘Paternity guardianship is not about mothers, it’s about children’s rights. A child comes from a couple. However complex adult relationships can be, children’s rights must be guaranteed’ (Fieldnotes, August 2013). In doing so, PU staff were enforcing a patriarchal and heteronormative policy. While most social workers identified as feminists, in practice they prioritised children’s rights over women’s personal interests.

Seeing pregnancy government through the lens of anticipation opens the path for a finer analysis of how foetuses are positioned as subjects in each context. Crucially, the case of the PU illustrates that anticipated foetal subjects can be objects of policy intervention and legal provisions even without a pro-life undertone, but rather through the ‘colonisation’ of pregnancy by children’s rights. Pregnant women thus come under surveillance just like mothers do, in a way that (unintentionally in this case) limits their autonomy and agency.

## Conclusion

Anticipation is a useful concept for drawing comparisons across the reproductive process, from preconception to parenting. The two dimensions introduced here – subjectification effects and future images – can be explored in relation to any reproductive stage and for comparing reproductive regimes over time and space. In the context of the PU, I described how anticipation positions pregnant women and foetuses in a constant dialectic with their possible futures. Anticipatory regimes have important implications for reproductive justice since anticipating future children’s rights during pregnancy tends to translate into the restriction of pregnant people’s autonomy to choose whether and how to carry their pregnancies to full term, and how to raise their children. However, anticipation and ideas about the future transcend the question of reproductive surveillance and control; these are core components of reproductive imaginaries (Dow, 2016; Franklin, 2013). As such, anticipation provides a unique lens across reproductive events that could foster transversal rather than compartmentalised understandings of the reproductive process, answering Almeling’s (2015) and Faircloth and Gürtin’s (2018) calls for more comprehensive approaches to reproduction.

The analysis of ‘future images’ pursues the endeavour of the sociology of reproduction to illustrate how reproduction is ‘stratified’ (Ginsburg and Rapp, 1995). Visions of the future reveal how a ‘good’ or ‘bad’ reproductive subject is conceived in a specific context and from there, how class, race, gender or sexuality influence who is empowered to reproduce and nurture their children and who is not. Looking at anticipatory regimes also highlights how pregnancy care seeks to normalise the future by re-orienting people’s behaviours. Normalisation operates both as a disciplinary power relation – at the individual level – and as a biopolitical project – in the public health effort to improve children’s health. The analysis of ‘future images’ in the PU revealed that it is not only pregnant bodies that are targeted but pregnant people’s minds and social lives – their conformity with the dominant norms of proper motherhood (Ballif, 2020). Analysing anticipatory regimes can thus further the understanding of the complex mechanisms that stratify reproduction.

Pregnancy represents a bridge between what is usually considered the domain of reproductive studies (fertility and conception), the anthropology of birth and parenting culture studies. This does not mean, however, that pregnancy should be considered to logically succeed conception and precede parenting. Being fertile or conceiving a child does not automatically result in full-term pregnancy, not all parenting experiences involve pregnancies, and all pregnancies do not result in a person becoming a parent. Reproduction tends to be thought of along a teleological model, with the ‘normal’ outcome of pregnancy considered to be the live birth of a baby, and where foetuses are seen as potential humans – a model that could be named ‘reproductive teleology’ as an expansion of Franklin’s (1991: 196) ‘fetal teleology’. If connections must be made across reproduction and parenting, they must not overshadow the multiplicity of reproductive experiences by duplicating reproductive teleology. The aim of such an endeavour would instead be, in my view, to gain a more global picture of the challenges to reproductive justice over time and around the world.

## Notes

1. Representations and experiences of pregnancy can differ significantly according to different cultural and national contexts (Ivry, 2010; Jordan, 1978; Morgan, 2006; Paxson, 2004) but also along race, class, gender and sexuality lines (Bridges, 2011; Mendes, 2021; Walks, 2015). I focus here on the dominant representations of reproduction and parenting that are associated with white, middle-class, heterosexual, Euro-American practices, first because these have been most described in the literature; second, because they are hegemonic and thus also have a major impact on marginalised populations; and third, because this population formed the majority of clients in the Perinatal Unit.

2. I am mostly using the term ‘pregnant *women*’ in this article – and not ‘persons’ – because this is how pregnant subjects are imagined and named in the PU. The fact that this language excludes trans, queer or non-binary persons was reflected by the absence of these persons in the unit. Most scholars in the field also write about pregnant persons as ‘women’. When I offer more general remarks in the conclusion, I use ‘pregnant persons’ to reflect my concern with the exclusionary effects of language.

3. The names of the unit, staff members and users have been replaced by pseudonyms.

4. In Switzerland, the monitoring of pregnancy is performed almost exclusively by gynaecologists, and prenatal care is paid for by (mandatory) health insurance (Burton-Jeangros et al., 2014; Manaï et al., 2010). Prenatal care and birth in general in Switzerland reflect prevailing trends towards medicalisation and risk logic (Hammer and Burton-Jeangros, 2013; Maffi and Gouilhers, 2019).

5. Since the research project did not aim to collect health-related personal data, it did not require authorisation from an ethical committee, according to the Swiss Federal Act on Research Involving Human Beings of 30 September 2011.

6. I have translated the data from French for this article.

7. It was common for PU staff to use ‘Baby’ as a proper noun in French as an affectionate way to personify the future child. This is not very common in French or in medical settings, where phrases such as ‘the baby’ or ‘your baby’ are usually preferred.

8. Article 309 of the Swiss Civil Code, in force until 1 July 2014: ‘As soon as an unwed pregnant woman requests it from child protection authorities or as soon as the latter have been informed of the birth, they name a legal guardian tasked with establishing paternal filiation, counselling and assisting the mother in an appropriate way’ (my translation).
